# Anisotropic Confinement, Electronic Coupling and Strain Induced Effects Detected by Valence-Band Anisotropy in Self-Assembled Quantum Dots

**DOI:** 10.1007/s11671-010-9786-8

**Published:** 2010-10-01

**Authors:** L Villegas-Lelovsky, MD Teodoro, V Lopez-Richard, C Calseverino, A Malachias, E Marega, BL Liang, Yu I Mazur, GE Marques, C Trallero-Giner, GJ Salamo

**Affiliations:** 1 Departamento de Física, Universidade Federal de São Carlos, São Carlos, SP 13565-905, Brazil; 2 Instituto de Física, Universidade Federal de Uberlândia, Uberlândia, Minas Gerais 38400-902, Brazil; 3 Arkansas Institute for Nanoscale Materials Science and Engineering, University of Arkansas, Fayetteville, AR 72701, USA; 4 Laboratório Nacional de Luz Síncrotron, Campinas, Brazil; 5 Instituto de Física de São Carlos, Universidade de São Paulo, São Carlos, SP 13560-970, Brazil; 6 Faculty of Physics, Havana University, 10400, Havana, Cuba; 7 Department of Electrical Engineer, University of California, Los Angeles, CA 90095, USA

**Keywords:** Molecular beam epitaxy, Self-assembled quantum dots, Inter-dot coupling, Anisotropic effects, Linear polarized photoluminescence emission, Grazing-incidence X-ray diffraction synchrotron, Optoelectronic

## Abstract

A method to determine the effects of the geometry and lateral ordering on the electronic properties of an array of one-dimensional self-assembled quantum dots is discussed. A model that takes into account the valence-band anisotropic effective masses and strain effects must be used to describe the behavior of the photoluminescence emission, proposed as a clean tool for the characterization of dot anisotropy and/or inter-dot coupling. Under special growth conditions, such as substrate temperature and Arsenic background, 1D chains of In_0.4_Ga_0.6_ As quantum dots were grown by molecular beam epitaxy. Grazing-incidence X-ray diffraction measurements directly evidence the strong strain anisotropy due to the formation of quantum dot chains, probed by polarization-resolved low-temperature photoluminescence. The results are in fair good agreement with the proposed model.

## Introduction

Recent attention has been given to the study of coupled quantum dot (QD) arrays for their potential application in quantum information processing [1-3]. The self-assembling process and its control become essential concerns in the search for new proposals of optoelectronic and quantum computing devices. Also, the spinor states in quasi-zero dimensional systems and their electronics have become features of renewed interest [4-7]. High uniformity of size, shape and distribution control of dot arrays are required in many application proposals like detectors, low-threshold lasers and photonic crystals. The lack of control over the self-assembly process of formation of these QDs leads to inhomogeneous broadening in size and/or shape that may degrade the quality of a device application. Therefore, the need for probing size, shape and effective inter-dot coupling has become an important area of research in recent years [8-12].

The anisotropy observed in linearly polarized PL-emissions from self-assembled QDs has been studied in recent years, and several works have detected some correlation with the anisotropic shape of the QD array [13-16]. There is also an agreement about the complexity of valence-band effects in QDs as a relevant issue when dealing with optical response from transitions between these completely localized states [7,17,18].

In the present work, we addressed mechanisms of testing simultaneously one-dimensional (1D) lateral ordering of dots, inter-dot coupling and 2D anisotropy of self-assembled QDs from studies of grazing-incidence X-ray diffraction (GID) and polarized photoluminescence (PL) emissions under different excitation power. This work has been motivated by the plausibility of controlled self-assembling growth of 1D dot arrays (QD chains) [19] and their potential use for testing important quantum effects such as correlation of information and optical coupling between dots where the relevant aspects of effects associated with inter-dot coupling and QD shape, size and distribution deserve special attention. It is also discussed the interplay between shape and strain fields with the inter-dot correlation that is revealed in the GID measurements and PL-emission spectra from QD arrays. Two sets of samples are investigated: one shows *chain-like* 1D correlation between neighboring dots and the other exhibits a mostly random island distribution. Two different QD shape models are used in order to calculate and test the polarized optical emission spectra dependence with spatial dot correlation and local geometry. The experimental confirmation included in this work highlights and supports the importance of probing correlated distribution in QD arrays for the characterization and improving of the growth-controlled processes.

## Theoretical Model

A multi-band **k** · **p** model based on the standard Kohn–Luttinger [20] and parabolic Hamiltonians to probe the electronic structure of holes and electrons, respectively, in dots grown along the [100] direction was developed. Due to strong valence-band admixture, such a procedure provides straightforward information on the relaxation of the inter-band optical transition selection rules, using lower computational efforts than in tight-binding calculation model, for example [13,14]. The built-in strain field distribution, which lead to the formation of self-assembled QD arrays, has been considered within the Bir–Pikus deformation potential model [21]. Uniform strain tensors are assumed, a model that neglects effects caused by variations at the QD interfaces [22,23]. This approximation works reasonably well for the study of ground-state properties of medium (~150 Å) and large (>250 Å) size dots.

**Figure 1:**
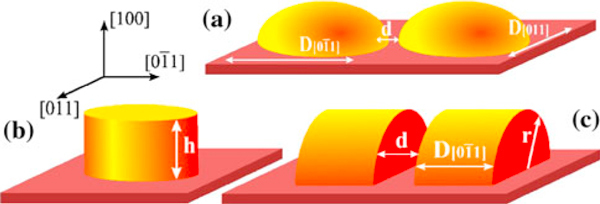
**(a) Schematic modeling of QD size and inter-dot coupling used in this study of self-assembled dots formed along the indicated crystalline directions**. (**b**) Confinement model for random distribution dots in the (100) plane. (**c**) Confinement model for testing anisotropic size and plausible inter-dot electronic coupling.

The double quantum dots structures under investigation are schematically illustrated in Figure 1. According to realistic dimensions the dots are assumed to have semi-cylindrical shape with radius ρ, laterally separated by an inter-dot barrier of thickness *d*. Since the main focus is concentrated in the tunneling along the lateral direction $\left[0\bar{1}1\right]$, the confining potential is defined as *V*(***ρ***, *z*) = *V*(***ρ***) + *V*(*z*), where the infinite barrier model have been used, as represented in Figure 1b (Figure 1c), at top (left) and bottom (right) interfaces, whereas the finite barrier model at the internal interfaces have been adopted, as represented in Figure 1c, in order to account for inter-dot coupling effects.

For the *GaInAs* alloys in consideration, at the center of the Brillouin zone, the split-off band is energetically well separated from the topmost valence subbands. In the limit of decoupled split-off band, the four-band Hamiltonian provides a good description of low-lying hole states by considering the coupling between the heavy-hole (*hh*) (*J* = 3/2, *j*_*z*_ = ±3/2) and the light-hole (*lh*) (*J* = 3/2, *j*_*z*_ = ±1/2). In the effective-mass approximation, when spanned in this basis, the kinetic energy of the hole is described by the 4 × 4 Kohn–Luttinger Hamiltonian

(1) $${ℋ}_{KL}=\frac{\hbar^{2}}{m_{0}}\left(\begin{array}{cccc} D_{hh} & A_{-} & 0 & {ℬ}_{-}\\ A_{-}^{*} & D_{lh} & {ℬ}_{-} & 0\\ 0 & {ℬ}_{-}^{*} & D_{hh} & A_{+}\\ {ℬ}_{-}^{*} & 0 & A_{+}^{*} & D_{lh} \end{array}\right)$$

where,

(2) $$\begin{array}{l} D_{hh}=-\left(\frac{\gamma_{1}+\gamma_{2}}{2}\right)\{{\hat{k}}_{+},{\hat{k}}_{-}\}-\left(\frac{\gamma_{1}-2\gamma_{2}}{2}\right){\hat{k}}_{z}^{2},\\ D_{lh}=-\left(\frac{\gamma_{1}-\gamma_{2}}{2}\right)\{{\hat{k}}_{+},{\hat{k}}_{-}\}-\left(\frac{\gamma_{1}+2\gamma_{2}}{2}\right){\hat{k}}_{z}^{2},\\ A_{\pm }=\mp \sqrt{3}\gamma_{3}{\hat{k}}_{\pm }{\hat{k}}_{z},\\ {ℬ}_{\pm }=-\frac{\sqrt{3}}{2}\frac{\gamma_{2}+\gamma_{3}}{2}{\hat{k}}_{\pm }^{2}, \end{array}$$

with the Luttinger parameters γ_*i*_ (*i* = 1, 2, 3), and the momentum operators k^±=k^x±ik^y,k^=−i∇.

The Hamiltonian of the hole in the quantum dot system is

(3) $$ℋ={ℋ}_{KL}+{ℋ}_{BP}+V(\rho )+V(z)$$

where *V*(***ρ***) is an infinite barrier outside of the semi-cylindrical cross-section, and *V*(*z*) is a double quantum well potential with infinite high outside walls, whose finite barrier is due to the offset between the band edges in the well and barrier materials; ℋ_*BP*_ is the Bir–Pikus Hamiltonian [21].

By exploring cylindrical symmetry in the Kohn–Luttinger model, the wave function of a hole state can be written in the form

(4) $$\Psi_{v}=\sum_{j,n,m,\alpha }C_{j}^{n,m}(\alpha )F_{j}(z)f_{n,m}(\rho ,\varphi )|\alpha \rangle .$$

The indexes (*j*, *n*, *m*) label the quantization along the *z*-direction (*j*) and in-plane (*n*, *m*) quantum numbers, respectively, α denotes the *spin-up* (| ↑ >) and *spin-down* (| ↓ >) periodic Bloch function character, namely: |*hh* ↑⟩, |*lh* ↑⟩, |*hh* ↓⟩ or |*lh* ↓⟩ and, finally, $C_{j}^{n,m}(\alpha )$ are the weight coefficients in the basis set of envelope wavefunctions, *F*_*j*_(*z*)*f*_*n,m*_(*ρ*, *φ*), at a position (***ρ***, *φ*) inside the dot. The solutions for the in-plane motion, *f*_*n,m*_(*ρ*, *φ*), are given by [24]

(5) $$\begin{array}{l} f_{n,m}(\rho ,\varphi )=\frac{2}{a\sqrt{\pi }|J_{n+1}(\mu_{n,m})|}J_{n}\left(\mu_{n,m}\frac{\rho }{a}\right)\sin(n\varphi ),\\ \;n=1,2,..., \end{array}$$

for semi-cylindrical confinement (Figure 1c). In these expressions, μ_*n*,*m*_ is the *m*th zero of the Bessel function of order *n*, *J*_*n*_(*x*), whereas the form of function *F*_*j*_(*z*) depends on the profile potential along *z*-direction between the dots. The depth of the quantum well is determined by the offset between the valence-band edges in the dot and the barrier materials. For the *GaAs*/*In*_0.4_*Ga*_0.6_*As* interface, the valence-band offset can be estimated as Δ*E*_*v*_ = 214 meV. By analytically solving the Schrödinger equation for holes and regarding the mismatch between the Luttinger parameters in the *GaAs*/*In*_0.4_*Ga*_0.6_*As* interfaces, the transcendental equation is derived, which determines all subband energies (*j*) and the corresponding wavefunctions (see Appendix 1). The signal (±) in the Eq. 16 provides them, respectively, with symmetric or asymmetric character $F_{j}^{\pm }(z)$. Taking advantage of this fact, the Hilbert space for the hole wavefunctions Ψ_*v*_(**r**) can be split into two orthogonal subspaces, labeled *I* and *II*, that are classified according to the parity of the quantum number *j*. As a result, the Hilbert subspace *I*(*II*) gathers spinor states with *spin-up* (*spin-down*) components having *odd j*-values (*even j*-values) that are coupled with states with *spin-down* (*spin-up*) and *odd j*-values (*even j*-values). Hence, the eigenvalue problem for the Hamiltonian in Eq. 3 can be solved independently for each class of states *I* and *II*. The hole state wavefunction (4) for a given subspace can then be written as

(6) $$\Psi_{v}^{I(II)}=\sum_{j,n,m}\left(\begin{array}{c} C_{2j-1(2j)}^{n,m}(hh↑)F_{2j-1(2j)}(z)f_{n,m}(\rho ,\varphi )|hh↑\rangle \\ C_{2j(2j-1)}^{n,m}(lh↑)F_{2j(2j-1)}(z)f_{n,m}(\rho ,\varphi )|lh↑\rangle \\ C_{2j(2j-1)}^{n,m}(hh↓)F_{2j(2j-1)}(z)f_{n,m}(\rho ,\varphi )|hh↓\rangle \\ C_{2j-1(2j)}^{n,m}(lh↓)F_{2j-1(2j)}(z)f_{n,m}(\rho ,\varphi )|lh↓\rangle \end{array}\right).$$

The hole states of the semi-cylindrical QDs system are calculated by exact diagonalization of the Hamiltonian ℋ, on a finite basis set expansion given by Eq. 6 using a standard numerical diagonalization technique. The matrix elements of the momentum operators ${\hat{k}}_{\pm }^{2},\;{\hat{k}}_{\pm }$ and ${\hat{k}}_{z}$ involved in the off-diagonal terms Eq. 2 of the Hamiltonian ℋ_*KL*_ are given in Appendix 2.

**Figure 2:**
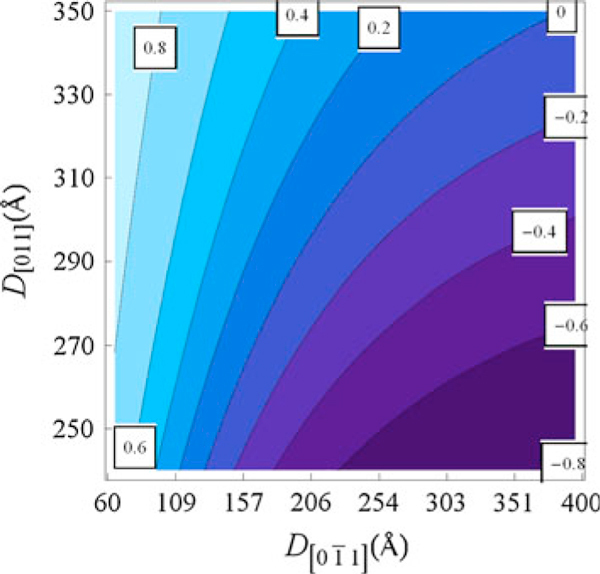
**Oscillator strength contours ${\text{Pol}}_{\left[011\right]}-{\text{Pol}}_{\left[0\bar{1}1\right]}$ for *correlated* dots with inter-dot distance *d* = 160 Å and strain order factor ε_||_ = –0.3%**.

As shown schematically in Figure 1, effects associated with isotropic and anisotropic spatial confinements are simulated in the calculation by changing the lateral sizes, *D*_011_ and $D_{\left[0\bar{1}1\right]}$, in the (100) plane as well as the inter-dot distance (*d*). Two geometry cases will be studied: (i) *Uncorrelated dots*, which consider isotropic spatial confinement in the (100) plane, with $D_{011}=D_{\left[0\bar{1}1\right]}$, without inter-dot coupling. The spin quantization axis (*z*-axis) is chosen along direction [100] (Figure 1a) and 2D dot distribution is random; (ii) *Correlated dots*, which consider anisotropic spatial confinement ($D_{\left[011\right]}\neq D_{\left[0\bar{1}1\right]}$) and include inter-dot coupling (Figure 1b) that leads to a *chain-like 1D* dot alignment. Here, the spin quantization axis (*z*-axis) must be set along the $\left[0\bar{1}1\right]$ direction [25,26].

These two models were tested and compared in order to search for the main qualitative differences between optical emission probabilities for light polarized along and perpendicular to the *z*-axis, respectively. This modeling tests the different behavior of optical emissions associated fundamentally with the difference between heavy-hole (*hh*) and light-hole (*lh*) longitudinal and transversal ellipsoidal effective masses as well as the effects originated from the strain fields on these hole energy levels.

The oscillator strength for optical electric fields linearly polarized along the $\left[0\bar{1}1\right]$ and parallel to [011] directions (see Figure 1) can be calculated as ${\text{Pol}}_{\left[011]([0\bar{1}1]\right)}={\left|\langle \Psi^{\text{cond}}\left|\vec{p}\cdot {\hat{e}}_{\left[011]([0\bar{1}1]\right)}\right|\Psi^{\text{val}}\rangle \right|}^{2}$. For *uncorrelated dot* arrays, showing mostly random distribution (case (i)), they are given by

(7) $$\begin{array}{l} {\text{Pol}}_{\left[011]([0\bar{1}1]\right)}=2|-\frac{C_{1}^{1,0}(hh↑)}{\sqrt{2}}L_{hh}^{1}+\frac{C_{1}^{1,0}(lh↓)}{\sqrt{6}}L_{lh}^{1}\\ \;\;{-(+)i\frac{C_{1}^{1,0}(hh↑)}{\sqrt{2}}L_{hh}^{1}-(+)i\frac{C_{1}^{1,0}(lh↓)}{\sqrt{6}}L_{lh}^{1}|}^{2}P^{2}, \end{array}$$

where *P* = ⟨*s*|*p*_*x*_|*x*⟩ = ⟨*s*|*p*_*y*_|*y*⟩ = ⟨*s*|*p*_*z*_|*z*⟩ is the isotropic conduction-valence-band momentum matrix element between functions at the Γ-point, $L_{hh(lh)}^{j}=\int F_{j}^{\text{cond}}(z)F_{j}^{hh(lh)}(z)\text{d}z$ is the overlap between *j*th electron and hole envelope functions along *z*-axis, and the factor 2 is due to double spin degeneracy.

All coefficients $C_{j}^{n,m}(\alpha )$, shown in Eq. 7, are real when calculated for cylindrical *uncorrelated dot* array case, and using the expansion set in Eq. 3. This result leads to identical oscillator strengths and, consequently, equal PL intensities for both optical linear polarizations. More specifically,

(8) $${\text{Pol}}_{\left[011\right]}={\text{Pol}}_{\left[0\bar{1}1\right]},$$

according to Eq. 4, and this identity is independent of QD size. Besides, neither hydrostatic nor axial strain contributions would induce changes to Eq. 8 in this symmetric case (unless anisotropic strains are applied). Therefore, a distribution of cylindrical *uncorrelated dots* over the (100) plane would lead to identical linear PL-emission intensities polarized along and perpendicular to the *z*-axis.

In *correlated arrays* showing preferential dot diffusion, the compressive strain can be relaxed by forming *1D* arrangement, as occurring for strain distribution in free-standing superlattices. In this case, the in-plane strain is defined by ε_||_ = ε_xx_ = ε_yy_ = (*a*_⊥_ - *a*_*w*_)/*a*_*w*_, where the lateral lattice constant (*a*_⊥_) can be calculated as [27]

(9) $$a_{⊥}=\frac{a_{w}L_{w}/S_{w}a_{w}^{2}+a_{b}L_{b}/S_{b}a_{b}^{2}}{L_{w}/S_{w}a_{w}^{2}+L_{b}/S_{b}a_{b}^{2}}.$$

Here, *S*_α_ = (*S*_11_ + *S*_12_)_α_ is the sum of elastic compliance constants, *L*_α_ (*a*_α_) is the width (*bulk* lattice constant) of the corresponding layers regions α = *w* (well) or *b* (barrier). In this way, a 3% strain can be relaxed to a value near 1%. Although shear strain contribution, which affects the separation between *hh* and *lh* subbands, becomes relaxed, the hydrostatic strain component leads to the effective reduction of the inter-dot potential barrier, which enhances the inter-dot coupling and tunneling. The envelope function spreading along the direction $\left[0\bar{1}1\right]$ favors the confinement of a carrier with higher in-plane effective mass, which leads to the exchange of the ground-state character, since $m_{lh}^{\left[0\bar{1}1\right]}>m_{hh}^{\left[011\right]}$.

The effects associated with the anisotropic confinement, within the inter-dot coupled model and simulated by a semi-cylindrical dot shape (see Figure 1c), uses only the subset of the expansion functions in Eq. 5 that complies with *null* boundary conditions at the flat part of the semi-cylinder. The corresponding linear crossed polarized optical matrix elements, for this *correlated dot* array model (case (ii)), are given by

(10) $${\text{Pol}}_{\left[0\bar{1}1\right]}=2\left\{{\left|\sqrt{\frac{2}{3}}C_{1}^{1,0}\left(hh↑\right)L_{hh}^{1}\right|}^{2}+{\left|\sqrt{\frac{2}{3}}C_{2}^{1,0}\left(hh↓\right)L_{hh}^{2}\right|}^{2}\right\}P^{2},$$

(11) Pol[011]=2{|C11,0(hh↑)2Lhh1−C11,0(lh↓)6Llh1|2+|C21,0(hh↓)2Lhh2−C21,0(lh↑)6Llh2|2}P2.

Here, the factor 2 occurs due to the summation over subbands *j* = 1,2 since these states are nearly degenerate for large inter-dot separation, *d*. It is clear that the identity in Eq. 8 has changed and no longer holds for all values of the inter-dot distance and QD sizes. We will be showing below that mass anisotropy of hole ground-state might be hold responsible for these anisotropic optical emission intensities once the dot confinement strength becomes relaxed in certain directions, whether by dot size anisotropy and/or by inter-dot coupling tuned by the strain fields.

First of all, let us analyze the effect of the spatial confinement in the case of a single dot with the semi-cylindrical shape, namely: the limiting case *d* → ∞ shown in Figure 1c. As the strength of the spatial confinement is relaxed along the direction $\left[0\bar{1}1\right]$ by the QD size increase, the topmost valence band becomes occupied by a state with a stronger *lh*-character and reduced *hh*-contribution [4,28,29]. This effect is caused by the strong hole mass anisotropy, namely: $m_{hh}^{\left[0\bar{1}1\right]}>m_{lh}^{\left[011\right]}$ while $m_{lh}^{\left[0\bar{1}1\right]}>m_{hh}^{\left[011\right]}$. It can be noted, from simple arguments, that *hh*- or *lh*-mass character of the valence-band ground-state can be interchanged by weakening the spatial confinement strength in the direction $\left[0\bar{1}1\right]$. Under weak confinement regime, the total energy determining the level position is mainly inversely proportional to the effective mass, as

(12) $$E^{hh}\propto \frac{1}{m_{hh}^{\left[0\bar{1}1\right]}{\langle D_{\left[0\bar{1}1\right]}\rangle }^{2}}+\frac{1}{m_{hh}^{\left[011\right]}{\langle D_{\left[011\right]}\rangle }^{2}},$$

and

(13) $$E^{lh}\propto \frac{1}{m_{lh}^{\left[0\bar{1}1\right]}{\langle D_{\left[0\bar{1}1\right]}\rangle }^{2}}+\frac{1}{m_{lh}^{\left[011\right]}{\langle D_{\left[011\right]}\rangle }^{2}},$$

where ⟨*D*_[011]_⟩ and $\langle D_{\left[0\bar{1}1\right]}\rangle$ denote mean confining lengths. Consequently, by tuning the confinement anisotropically, the condition *E*^*lh*^ <*E*^*hh*^ can be attained due to the mass anisotropy of carriers. As a result, the corresponding envelope functions must be more extended in one direction than the other. Thus, the corresponding PL transitions allowed for certain light polarization can probe the anisotropic character of the Bloch functions that, in the multi-band calculations, are determined by the values of the expansion coefficients in Eq. 4. It is noted, from Eq. 10, that a state having small *hh*-character and, consequently, small values of coefficients $C_{1}^{1,0}(hh↑)$ and $C_{2}^{1,0}(hh↓)$, produces smaller oscillator strength for optical transition polarized along the inter-dot coupling direction $\left[0\bar{1}1\right]$.

Figure 2 shows the oscillator strength values calculated for two coupled semi-cylindrical QDs with two values of the transverse diameter, *D*_[011]_, as a function of the axial length, $D_{\left[0\bar{1}1\right]}$ (see Figure 1c). Here, we have estimated the strain strength to hold with the *uncorrelated dot* array condition $D_{\left[0\bar{1}1\right]}=D_{\left[011\right]}$ and confirm that the bigger the transverse size of dot array is (Figure 1b) the smaller must the strain order factor be. Furthermore, for compressive strain ε_||_ > ε_⊥_, the crossing point ${\text{Pol}}_{\left[0\bar{1}1\right]}={\text{Pol}}_{\left[011\right]}$ can be shifted toward the dotted line. For dilation strain, with ε_||_ < ε_⊥_, the crossing point is shifted away from the *uncorrelated dot* condition, and this condition can be attained in self-assembled QDs grown along the [100] direction. Certainly, shear strain field distribution is able to tune the equal oscillator strength condition for these mutually perpendicular polarized emissions in isolated anisotropic QDs.

**Figure 3:**
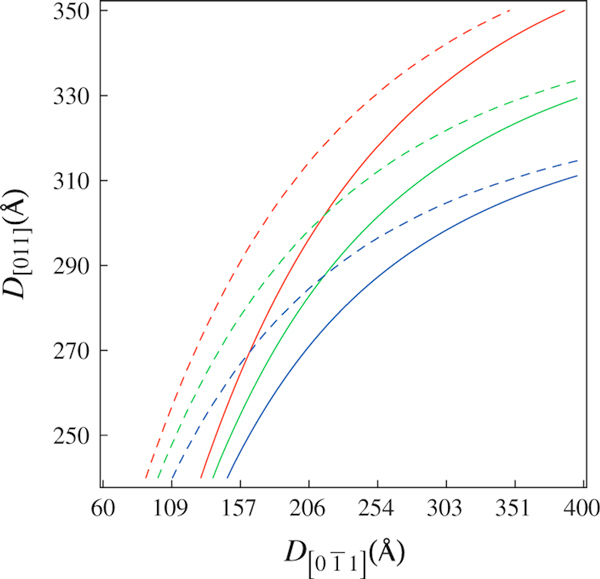
**Oscillator strength contours fulfilling ${\text{Pol}}_{\left[011\right]}={\text{Pol}}_{\left[0\bar{1}1\right]}$ for *correlated* dots with inter-dot distances *d* = 160 Å (*solid line*) and 330 Å (*dashed line*) and strain order factors ε_||_ = –0.3% (*red*), ε_||_ = –0.4% (*green*) and ε_||_ = –0.5% (*blue*)**.

Analogously to the exchange of ground-state character induced by anisotropic confinement and shear strain fields, this effect can be also produced by electronic coupling between nearest-neighboring QDs, an effect that leads to the enhancement of the effective value $\langle D_{\left[0\bar{1}1\right]}\rangle$. The interchange of ground-state character is highly favored in coupled dots by increasing the inter-dot tunneling, as can be seen in Figure 3, which leads to the envelope function spreading along the coupling direction, $\left[0\bar{1}1\right]$. In order to show this effect, we have used the combination of dots with finite inter-dot separation, *d*. Note, in Figure 3, that coupled dots will show a left-shifted crossing point for equal oscillator strength, when compared to the *uncorrelated* dot case. As discussed before, this shift can be further modified by shear strain fields.

**Figure 4:**
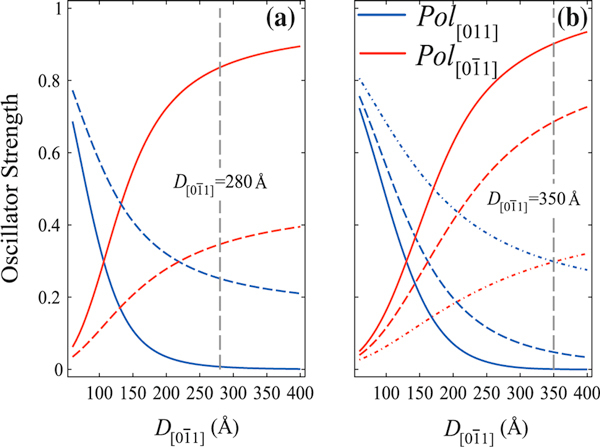
**Calculated oscillator strengths for crossed linear optical polarizations along the directions $\left[0\bar{1}1\right]$ (*red line*) and [011] (*blue line*) for two coupled QD's (Figure 1c) with semicylindrical shape and axis in the $\left[0\bar{1}1\right]$ direction with (a) $D_{\left[0\bar{1}1\right]}$ = 280 Å, *d* = 160 Å upon strain order of ε_||_ = - 0.3% (*solid line*) and -0.9% (*dashed line*). (b) $D_{\left[0\bar{1}1\right]}$ = 350 Å, *d* = 330 Å and ε_||_ = - 0.1% (*solid line*), -0.2% (*dashed line*) and -0.3% (*dashed-dotted line*)**. The crossing point stands for isotropic optical emission.

**Figure 5:**
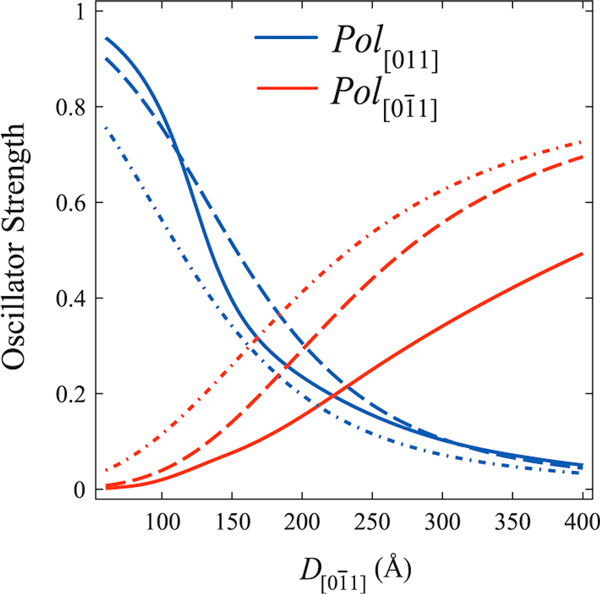
**Calculated oscillator strengths for crossed linear optical polarizations for a strained system of two coupled QDs with different inter-dot distances *d* = 160 Å (*dashed line*), 330 Å (*dashed-dotted line*) and infinite (*solid line*) corresponding to a single (isolated) QD**. Here was taken a lateral size *D*_[011]_ = 350 Å and a strain order factor ε_||_ = - 0.2%.

For the limiting cases (see Figure 4), $D_{\left[0\bar{1}1\right]}\ll D_{\left[011\right]}$ and $D_{\left[0\bar{1}1\right]}\gg D_{\left[011\right]}$, the oscillator strengths for polarized emissions attain the conditions ${\text{Pol}}_{\left[0\bar{1}1\right]}\ll {\text{Pol}}_{\left[011\right]}$ and ${\text{Pol}}_{\left[0\bar{1}1\right]}\gg {\text{Pol}}_{\left[011\right]}$, respectively, and these results are attributed to the anisotropy of hole effective masses. The crossing point where the polarized emissions have equal intensities can be shifted by the shear strain contribution to *hh*- and *lh*-energy level positions. In Figure 5, it can be observed that the crossing points are shifted to the right as the strain order factor and/or inter-dot distance are increased. Furthermore, two asymptotic limits $D_{\left[0\bar{1}1\right]}\approx D_{\left[011\right]}$ > 400 Å and $D_{\left[0\bar{1}1\right]}\approx D_{\left[011\right]}$ < 200 Å where the crossing points coincide were found out, respectively, for various strain strengths and for different inter-dot distances.

## Experimental Confirmation of the Purposed Theory

Experiments that confirm this modeling were performed using *In*_0.4_*Ga*_0.6_*As* QDs grown by molecular beam epitaxy on semi-insulating (100)*GaAs*. The QDs were obtained using the Stranski–Krastanov growth mode. Two set of samples were prepared for the experiments: (A) QDs with strong anisotropy in shape along $\left[0\bar{1}1\right]$ direction and with partial ordering along that; (B) QDs with weak or no anisotropy on the (100) surface and large separation in both in-plane directions. The shape and the distribution of QDs were controlled by the Arsenic background. The use of *As*_2_ or *As*_4_ background during the growth allows the control of group III element diffusion on *GaAs* (100) surfaces, providing choices for different dot samples with the same composition but different shapes and distribution. Details of growth mechanisms and the processes involved in diffusion controlling by the background Arsenic environment are described in Ref. [19].

**Figure 6:**
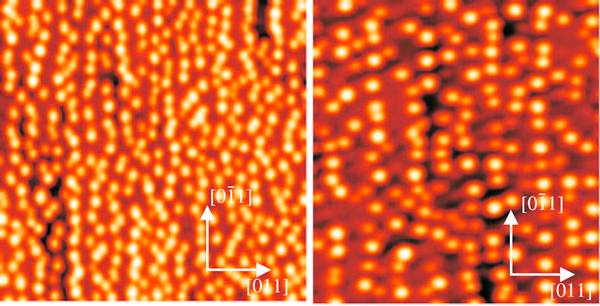
**One layer AFM 1 × 1 μm image of *In*_0.4_*Ga*_0.6_*As* QDs in samples grown under different conditions**. Sample A (*left*) shows 1D *chain-like* ordering along the $\left[0\bar{1}1\right]$ direction. Sample B (*right*) shows mostly isotropic or randomized dot distribution in the (001) plane.

Two sets of samples A were grown under *As*_4_ background. In one set, the layer of dots was left uncapped for morphology analysis, and in the other, the QDs were buried with *GaAs* for low-temperature PL analysis. The other two sets samples B were grown under the same conditions as the sets A, except that under *As*_2_ background. Surface morphologies of the two uncapped samples were performed by using atomic force microscopy (AFM), as shown in Figure 6, imaged by Nanoscope IV in the tapping mode and using a high-resolution Silicon tip. The (1 × 1) μm AFM images show the morphologies of the *In*_0.4_*Ga*_0.6_*As* uncapped dot samples. The mean dot size and the center-to-center distance along the $\left[0\bar{1}1\right]$ direction of both sets are displayed in Table 1. The AFM pictures show clearly the effect of different Arsenic background both on dot formation and distribution. The predominantly anisotropic dot shape and distribution obtained along $\left[0\bar{1}1\right]$ direction is for samples grown under *As*_4_ environment. Finally, these sets of samples enable us to use sample (B) as the reference for uncoupled QD arrays with mostly isotropic distribution on the (100) plane.

**Table 1:** Average QD parameters with dispersion obtained from a Gaussian fit of the AFM data

Sample	**Density (cm**^**-2**^**)**	$D_{\left[0\bar{1}1\right]}$**(Å)**	***D***_**[011] **_**(Å)**	*h* (Å)	*d* (Å)
A	3.9 × 10^10^	280 ± 12	220 ± 10	63 ± 12	160 ± 30
B	1.9 × 10^10^	350 ± 15	350 ± 15	90 ± 15	330 ± 75

$D_{\left[0\bar{1}1\right]}$: Dot width along $\left[0\bar{1}1\right]$; *D*_[011]_: dot width along [011]; *h*: QD height; *d*: inter-dot distance along the "chain-direction" $\left[0\bar{1}1\right]$

Grazing-incidence X-ray diffraction (GID) measurements were performed in both samples at the XRD2 beamline of the Brazilian Synchrotron Light Laboratory (LNLS), using a 4 + 2 axis diffractometer. The X-ray photon energy was fixed to 10 keV. Since both samples were capped by a *GaAs* 50 nm layer, the incident angle was fixed at 0.28°, slightly above the *GaAs* critical angle, maximizing the signal from the buried quantum dots. The diffracted signal was measured by integrating the exit angle from 0 to 1.2° [30].

**Figure 7:**
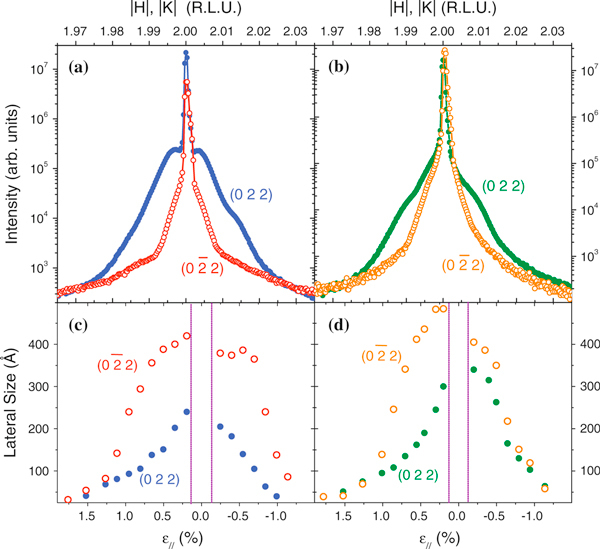
**Radial scans at the vicinity of the *GaAs* (022) (*solid dots*) and $\left(0\bar{2}2\right)$ (*open dots*) reflections for sample A (a) and B (b)**. Lateral size from iso-strain regions in samples A (**c**) and B (**d**) obtained from the width of transversal scans.

Figure 7a and 7b show longitudinal *θ* - 2*θ* scans in the vicinity of the in-plane (022) and $\left(0\bar{2}2\right)$ reflections for samples A and B, respectively. Such scans are sensitive to the strain relaxation inside the *In*_0.4_*Ga*_0.6_*As* QDs and *GaAs* surrounding lattice. For all scans, diffuse intensity is observed surrounding the narrow and intense *GaAs* Bragg peak, located at |*H*| = |*K*| = 2. For sample A, the longitudinal scan performed at the vicinity of the *GaAs* (022) reflection exhibits a much broader profile than the scan measured with the sample rotated by 90°, close to the ${}_{GaAs(0\bar{2}2)}$ reflection. Such a behavior indicates that a more effective strain relaxation for the islands may take place along the [022] direction, while a more strained lattice profile is found along the $\left[0\bar{2}2\right]$ direction. The intensity distribution in both profiles of Figure 7a is almost symmetric with respect to the *GaAs* peak position, denoting the existence of compressively strained *InGaAs* inside the QDs, as well as on the *GaAs* matrix surrounding the QDs [31]. Similar diffraction profiles are observed in the longitudinal scans performed on sample B (Figure 7b). For this sample, the difference of widths of diffuse intensity on (022) and $\left(0\bar{2}2\right)$ scans is not as pronounced as observed for sample A, indicating a less anisotropic relaxation.

In order to quantify the strain relaxation inside QDs in both samples, transversal scans were performed at several positions along the longitudinal profiles shown in Figure 1a and 1b. These scans (not shown here) are measured by fixing the θ - 2θ condition and varying the sample rotation angle θ solely. In momentum transfer space, the angular momentum transfer *q*_*a*_ = (4π/λ)sin(2θ/2)sing(Δθ) is varied, where Δθ = (θ/2θ)/2. Such a procedure allows to obtain the average lateral size *L* of regions inside the QDs with constant strain status by evaluating the width Δ*q*_*a*_ of transversal scans, *L* = 2π/Δ*q*_*a *_[30,32]. Values obtained for the local lateral size of iso-strain regions as a function of the in-plane strain status for samples A and B in the [022] and $\left[0\bar{2}2\right]$ directions are shown in Figure 7c and 7d, respectively. For both samples, the lateral size of iso-strain regions along the QDs chain direction is larger than along the axis parallel to the chains. The ratio $L_{\left[0\bar{2}2\right]}/L_{\left[022\right]}$, which is a quantitative indicator of the anisotropic lattice relaxation inside QDs, is larger for sample A than for sample B, corroborating the qualitative information inferred from the widths of longitudinal scans.

**Figure 8:**
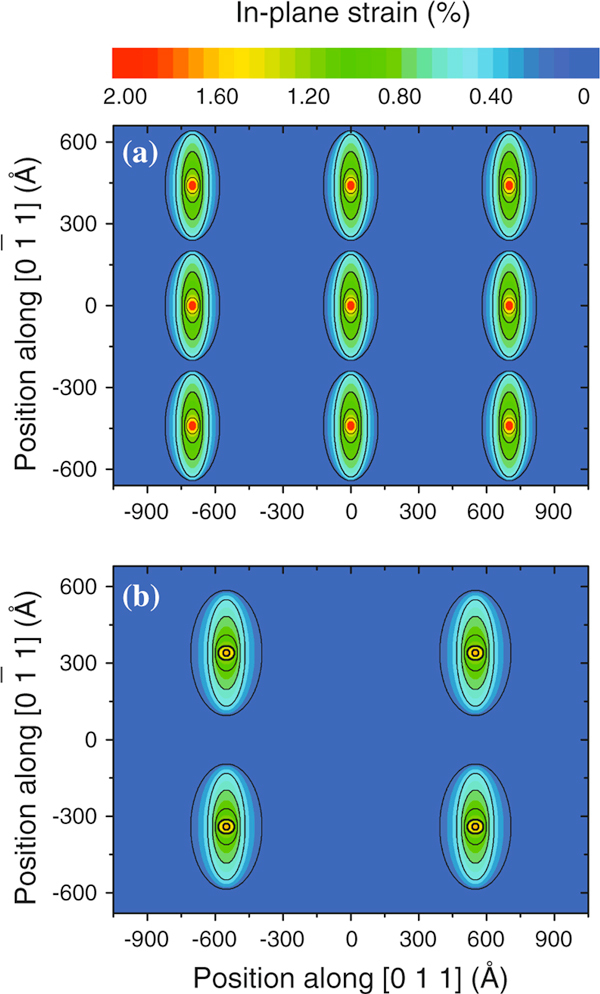
**In-plane projection of iso-strain regions for a field of view with several islands for samples A (a) and B (b)**. The in-plane strain represented in the color scale is relative to the GaAs bulk lattice.

Some considerations must be drawn before extending the analysis of the data shown in Figure 7c and 7d. For uncapped QDs, the relaxation of lattice parameter is monotonic from their base to their apex [32]. Capped QDs, in contrast, exhibit a non-monotonic gradient, with lateral and vertical strain variations. This condition generally implies in the existence of similar in-plane strain status on the island base and apex, both in contact with the *GaAs* matrix. It is therefore impossible to resolve vertically the position of iso-strain areas for the capped QDs with our GID measurements. Nevertheless, the lateral sizes observed represent a good approximation of the in-plane area of iso-strain regions projected on the substrate surface plane. Such approach allows for a visualization of the anisotropic strain relaxation. Since the diffraction signal observed at (|*H*|, |*K*|) > 2 is related to the existence of compressively strained *GaAs* surrounding the QDs [31], maps with the projected view of iso-strain areas were extracted from the experimental data by taking into account the *L* values for (|*H*|, |*K*|) < 2, which are directly related to compressively strained *In*_0.4_*Ga*_0.6_*As* from the islands (Tensile strained *GaAs* at the bottom and at the apex of the island also contribute to the diffracted intensity at |*H*| = |*K*| < 2. However, the total volume of material with local lattice parameter larger than *a*_*GaAs*_ outside the island is considerably smaller than the amount of material contained inside the islands. For a discussion on tensile strained substrate material see [33]). These projection maps for QDs from samples A and B are shown in Figure 8a and 8b, respectively. The iso-strain projection areas were drawn following the condition that they are contained on curves delimited by

(14) $$\frac{x^{2}}{L_{\left[022\right]}^{2}}+\frac{y^{2}}{L_{\left[0\bar{2}2\right]}^{2}}\leq 1,$$

where *x* and *y* are the in-plane coordinates along the [011] and $\left[0\bar{1}1\right]$ directions, respectively, considering the plane origin at the central QD position. Figure 8 shows the iso-strain areas for an in-plane region of approximately 1,100 Å × 700 Å, which contains 9 QDs for sample A and 4 QDs for sample B (see Table 1). The color scale in these maps refers to the in-plane strain with respect to bulk *GaAs*.

From Figure 8a, one clearly observes that iso-strain contour lines from one QD of sample A almost reach the neighbor QDs along the $\left[0\bar{1}1\right]$ chain direction. An asymmetric ratio $L_{\left[0\bar{2}2\right]}/L_{\left[022\right]}$ of 1.7 is found for the broader iso-strain contour lines of QDs in this sample, pointing out again to a more pronounced strain relaxation along the [011] axis. The physical presence of very close QDs along the chains may therefore induce a modulation of the strain field that allows for a gentle strain relaxation in the $\left[0\bar{1}1\right]$ direction. In sample B (Figure 8b), the asymmetric shape of iso-strain regions is still observed, but with a ratio $L_{\left[0\bar{2}2\right]}/L_{\left[022\right]}$ of 1.35. Although an elongation is observed along the $\left[0\bar{1}1\right]$ direction, the QDs are too apart from each other and do not strongly influence the strain field of the neighbor QDs in this direction.

**Figure 9:**
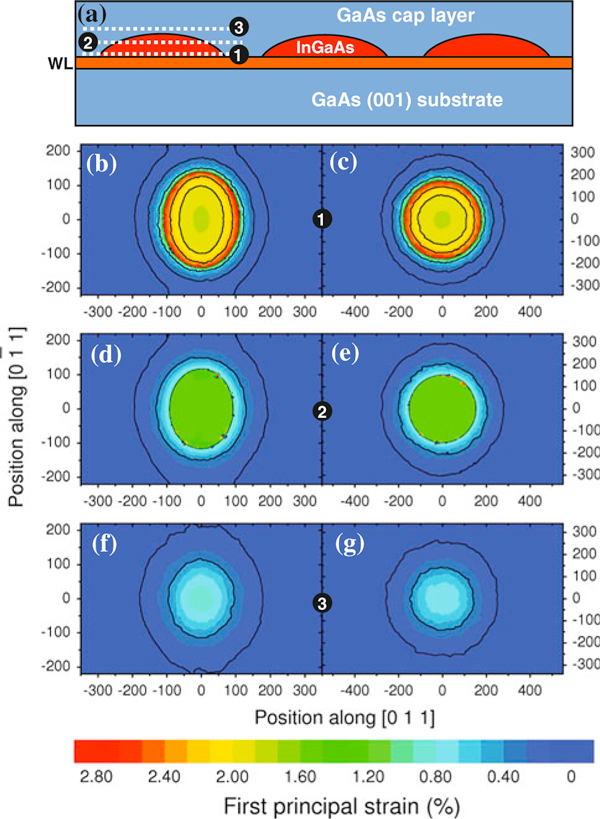
**(a) Representation of the two-dimensional cuts shown in maps panels (b–g) performed on the finite element method simulations with periodic contour conditions at the substrate box edges**. The color contours represent variations on the first axial principal strain, which allows a qualitative comparison with the GID data of Figure 8. Cuts on the bottom (**b**), middle (**d**) and top (**f**) of the average island of sample A show an elongated strain profile along the $\left[0\bar{1}1\right]$ directions. Similar cuts for the average island of sample B are seen on (**c**), (**e**) and (**g**).

Since the GID measurements do not reveal directly the height above the substrate of each iso-strain region finite element method, simulations were performed using a commercial software package to provide complementary information on the strain configuration of capped islands. In our simulations, a three-dimensional box containing a single *GaAs* capped *In*_0.4_*Ga*_0.6_*As* island was created for each sample, with periodic contour conditions at all lateral edges in order to take into account the symmetry of QD chains and the possible interaction with the strain field from neighbor QDs. A 15 Å thick wetting layer of nominal concentration was inserted between the islands and the substrate, following Ref. [34]. The island profiles used in this simulation were extracted from the AFM measurements in uncapped islands (Figure 6) that resulted in the dimensions from Table 1. The nominal composition was kept, assuming thus a negligible deviation of island stoichiometry from the nominal values (Anomalous grazing-incidence diffraction measurements performed at the *Ga* - *K* edge do not point out to deviations (within an error bar of 7%) from the nominal *In*/*Ga* content inside QDs.). Such assumptions consider that islands do not undergo dramatic changes in morphology or composition under capping, which is a valid approximation for the growth temperatures used here and the reduced strain with respect to pure InAs islands [35]. Finally, a 500 Å-thick cap layer was added to the simulation, as represented in Figure 9a.

Two-dimensional cuts of the simulated data are shown in Figure 9b, d, and 9f for sample A and Figure 9c, e, and 9g for sample B. The selected cuts are schematically depicted at Figure 9a and were chosen to be at the island bottom (b) and (c), middle (d) and (e), and top (f) and (g). Since the representation used in Figure 8 cannot be directly correlated to the Cartesian in-plane strain components *x* and *y*, the maps of Figure 9b–g were drawn as a function of the axial (first) principal strain component. Such principal component analysis allows for the reduction of the dimensionality of the data set, providing a resulting representation with radial symmetry. The axial strain component is given by [36]

(15) $$\varepsilon_{A}=\frac{\varepsilon_{xx}+\varepsilon_{yy}}{2}+\sqrt{{\left(\frac{\varepsilon_{xx}+\varepsilon_{yy}}{2}\right)}^{2}+\varepsilon_{xy}^{2}},$$

where ε_*xy*_ is the in-plane shear strain and ε_*xx*_, ε_*yy*_ the normal in-plane strains. For all principal strain maps, the color scale represents the deviation of the local lattice parameter with respect to the bulk local lattice parameter. Therefore, higher principal strain values are found in positions where the *In*_0.4_*Ga*_0.6_*As* lattice of the islands is fully strained to the *GaAs* lattice constant. Finite values of the axial strain component are also observed in regions surrounding the islands, in which the *GaAs* local lattice is affected by the proximity to the island. Selected contour level edges were marked by dark lines in all maps as a guide to the eyes.

The maps generated by in-plane cuts in the simulation of QDs in sample A clearly exhibit elongated contours along the $\left[0\bar{1}1\right]$ direction, most notably for the cuts at the island basis and middle. This indicates that for lower in-plane strain conditions, the lattice surrounding the islands behave as semi-continuous wires along the $\left[0\bar{1}1\right]$ direction. In the QDs of sample B, an elongation of axial strain contour levels is also observed along the chain directions for all maps. However, the anisotropic effect is much more reduced with respect to the results obtained for sample A.

**Figure 10:**
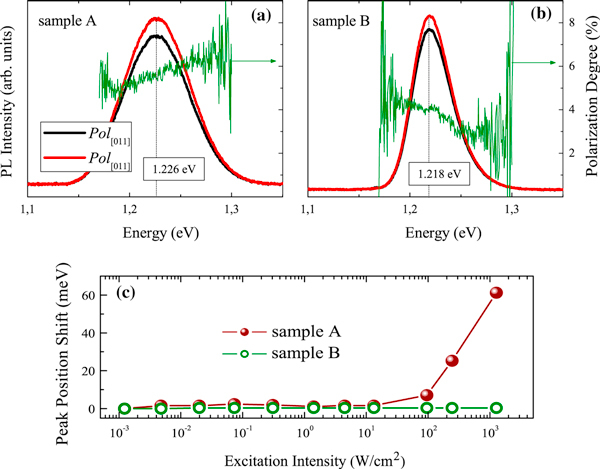
**PL spectra for crossed linear polarizations, taken at *T* = 10 K with excitation wave length λ = 532 nm along $\left[0\bar{1}1\right]$ and [011] directions for samples (a) A and (b) B**. The degree of linear polarization: $\left({\text{Pol}}_{\left[0\bar{1}1\right]}-{\text{Pol}}_{\left[011\right]})/({\text{Pol}}_{\left[0\bar{1}1\right]}+{\text{Pol}}_{\left[011\right]}\right)$ has been included in these panels. (**c**) PL peak position as a function of the excitation intensity.

The effects of different dot size distributions and inter-dot coupling have been analyzed by low-temperature linear polarized PL measurements carried out on the samples A and B buried with *GaAs*. The samples were placed in a closed-cycle Helium cryostat (Janis—CCS-150) and excited using a 532-nm continuous wave YAG laser (Coherent Verdi V10—10 W). The PL signal was carried out by a monochromator (SpectraPro 2500i—0.5 m focal length) and detected by a liquid-nitrogen-cooled *InGaAs* photodiode detector array (Princeton Instruments—model 7498-0001). Figure 10a and 10b show the PL intensity at 10 K for samples A and B, respectively, where the emission spectra, for each sample type, collected with two linear polarizations, namely: along $\left[0\bar{1}1\right]$ and along [011].

In Figure 10a, one may see a polarization degree around 6%, as might be expected due to the elongation in the quantum dots profile revealed by the AFM images (Figure 6) and strain distribution (Figure 8). As highlighted in Figures 2 and 3, the oscillator strength grows for emissions linearly polarized along the larger dot size direction. This behavior is enhanced for inter-dot separation up to *d* ~ 160 Å. When *d* is further reduced, the inter-dot tunneling probability increases considerably, and this behavior is also enhanced. The PL intensity polarized along coupling direction $\left[0\bar{1}1\right]$ is also enhanced in coupled QDs by the reduction of barrier heights due to hydrostatic strain of the order of 1%. Besides, the anisotropic PL-emissions from sample A, as shown in Figure 5a, can be qualitatively reproduced by the oscillator strengths, shown in Figure 3, calculated by using the nominal values for both samples. As seen in Figure 3, an effective increase in inter-dot tunneling (distance *d* ~ 160) Å would lead to the relaxation of the confinement along the $\left[0\bar{1}1\right]$ direction. These effects would lead to a hole ground-state character exchange from predominant-*hh* to -*lh*, and to the intensity difference between these cross-polarized emissions, experimentally confirmed by Figure 10a.

For the isotropic case, PL-emissions occur when $D_{\left[011\right]}≃D_{\left[0\bar{1}1\right]}$, a condition well-fulfilled for the cylindrical model of Figure 1b. By changing the dot shape and coupling along direction in (100) plane, the model shows that ${\text{Pol}}_{\left[011\right]}-{\text{Pol}}_{\left[0\bar{1}1\right]}$ condition can be obtained for semi-cylindrical geometry only for a small combination of values that emulates *uncoupled* dot distribution in the (100) plane if strain effects are included into the Hamiltonian. According to the theoretical modeling, an isotropic dot distribution on the (100) plane (case (i)) accounts for isotropic crossed polarized PL-emissions, as shown in Figure 5b for sample B. However, according to Figure 10b, a small polarization degree is still present in symmetric QDs, associated with the elongation that remains, as revealed by the Figure 8b. Such feature might come from the anisotropic diffusion rate of Indium atoms during the growth, which presents a higher mobility than the Gallium atoms. Furthermore, the Indium diffusion coefficient is faster along the $\left[0\bar{1}1\right]$ than along the [110] direction, and as a result, the quantum dots of sample B are not completely symmetric [37,38].

To confirm the results from X-ray measurements, Figure 10c displays the shift in peak position of the spectra as a function of the excitation intensity. Note, for sample A, a shift toward higher energies as the excitation intensity grows. Such a blue-shift for the elongated dots has been associated with the screening of the built-in electric field due to the presence of strain. On the other hand, for sample B no remarkable energy shift is observed showing that the strain is not so pronounced as in the previous case [39].

## Conclusions

The control and simulation of size anisotropy and effective inter-dot tunneling effects, as described in this work, is an important issue to be addressed during the characterization of ordered sets of coupled dots. The strain fields, present during the growth process of these QDs have led to the appearance of anisotropic geometric shapes, mostly elongated along the preferential direction. These effects can be probed by polarized optical responses from different samples. In summary, we have shown that the shape, spatial distribution and the inter-dot coupling of *InGaAs* self-assembled QDs can be probed and characterized by using linearly polarized PL-emissions. Valence-band effects due to admixing between hole states and strong anisotropic effective masses have led to different PL intensities in samples with lateral QD ordering forming "*chain-like*" structures. The envelope function model used here to describe the polarized optical responses showed fairly good agreement with structural AFM and X-ray data and may be used to predict or characterize the strength of inter-dot coupling and/or anisotropic dot shape and distribution.

## Appendix 1: Double Quantum Well Potential

After matching the wavefunctions fulfilling the hole-Shchrödinger equation at the interfaces using ∂_*z*_ ln *F*_*j*_(*z*|_*z*=*l*_ = β∂_*z*_ ln *F*_*j*_(*z*)|_*z*=*l*_ and ∂_*z*_ ln *F*_*j*_(*z*|_*z*=*l*+*d*_ = β^-1^∂_*z*_ ln *F*_*j*_(*z*)|_*z*=*l*+*d*_, we are able to obtain the transcendental equation

(16) $$\beta \frac{K}{k}\tan(kl)=-\tanh^{\pm 1}\left(\frac{Kd}{2}\right)$$

where $k=\sqrt{2m_{b}^{*}|E_{j}|}/\hbar$ and $K=\sqrt{2m_{w}^{*}|V-E_{j}|}/\hbar$ and $\beta =m_{b}^{*}/m_{w}^{*}$ is the rate between the hole effective masses in the barrier and the well region, respectively, $m_{b}^{*}$ and $m_{w}^{*}$, as well as *l* is the well width, *d* the inter-dot distance and *V* the barrier height. By carrying out numerical calculation, solutions of the Eq. 16 yields to the energy levels $E_{j}^{\pm }$ with the corresponding wavefunctions of the symmetric (+) and antisymmetric (-) hole states,

(17) $$F_{j}^{\pm }(z)=\{\begin{array}{ll} A^{\pm }(l,d)\text{sin}\left(kz\right), & \;\;\text{left well}\\ A^{\pm }(l,d)\text{sin}\left(kl\right)\frac{\text{exp}(K(z-l))\pm \text{exp}(-K(z-l)-d)}{1\pm \exp(Kd)}, & \;\;\;\text{barrier}\\ \pm A^{\pm }(l,d)\text{sin}\left(k(2l+d-z)\right), & \text{right well} \end{array}$$

where

$$\begin{array}{l} A^{+}(l,d)=2kK/(2kKl-K\;\sin\left(2kl\right)+k\;\sinh^{-2}\left(\frac{Kd}{2}\right)\\ \;\;\sin^{2}\left(kl\right)\left(\sinh\left(Kd\right)-Kd\right))\\ A^{-}(l,d)=2kK/(2kKl-K\;\sin(2kl)+k\;\cosh^{-2}\left(\frac{Kd}{2}\right)\\ \;\;\sin^{2}\left(kl\right)\left(\sinh\left(Kd\right)+Kd\right)) \end{array}$$

## Appendix 2: Matrix Elements

The matrix elements of the momentum operators are necessary in order to build the Hamiltonian matrix form of the ℋ_*KL*_. In polar coordinates the operators ${\hat{k}}_{\pm }$ are written as,

$${\hat{k}}_{\pm }=-ie^{\pm i\varphi }\left(\frac{\partial }{\partial \rho }\pm \frac{i}{\rho }\frac{\partial }{\partial \varphi }\right).$$

Projecting on the wavefunctions (5) $g_{t,t^{\prime }}^{\pm }\equiv a\langle t\left|{\hat{k}}_{\pm }\right|t^{\prime }\rangle$ with *t* = (*n*, *m*) and *t*' = (*n*', *m*'), it is straightforward to show that

(18) $$g_{t,t^{\prime }}^{+}=i\frac{n-n^{\prime }}{\left|n-n^{\prime }\right|}T_{n\pm 1,m^{\prime }}^{n,m};\;\text{if}\;n^{\prime }=n\pm 1,$$

with

$$T_{n^{\prime },m^{\prime }}^{n,m}=\frac{\mu_{n,m}/\mu_{n^{\prime },m^{\prime }}}{{\left(\mu_{n,m}/\mu_{n^{\prime },m^{\prime }}\right)}^{2}-1}\text{,}$$

and

(19) $$g_{t,t^{\prime }}^{+}=\frac{2n}{\mu_{t^{\prime }}\pi }\frac{1+{\left(-1\right)}^{n+n^{\prime }}}{J_{n^{\prime }+1}\left(\mu_{t^{\prime }}\right)J_{n^{\prime }+1}\left(\mu_{t}\right)}\times \zeta_{n^{\prime },m^{\prime }}^{n,m};\;\text{if }\;n^{\prime }\neq n\pm 1\text{,}$$

where $\zeta_{t^{\prime }}^{t}$ are numbers ruled by

(20) $$\zeta_{t^{\prime }}^{t}=\int_{0}^{\mu_{t^{\prime }}}\eta J_{n}\left(\frac{\mu_{t}}{\mu_{t^{\prime }}}\eta \right)\left[\frac{J_{n^{\prime }-1}\left(\eta \right)}{{\left(1-n^{\prime }\right)}^{2}-n^{2}}-\frac{J_{n^{\prime }+1}\left(\eta \right)}{{\left(1+n^{\prime }\right)}^{2}-n^{2}}\right]\text{d}\eta .$$

In the particular case where *t* = *t*' Eq. 20 can be reduced to $\zeta_{t}^{t}=0$

Also, it follows the relation

(21) $$g_{t,t^{\prime }}^{-}={\left(-1\right)}^{n^{\prime }+n+1}g_{t,t^{\prime }}^{+}\text{.}$$

The other matrix elements for the high-order operators ${\hat{k}}_{\pm }^{2}$ are evaluated numerically using Eqs. 18–21 and the matrix identity $\langle t\left|\hat{A}\hat{B}\right|t^{\prime }\rangle =\sum_{p}\langle t\left|\hat{A}\right|p\rangle \langle p\left|\hat{B}\right|t^{\prime }\rangle$.

It is worth to show that the element matrix of the diagonal terms in *D*_*hh*(*lh*)_ accomplished

$$\langle n,m|\{{\hat{k}}_{+},{\hat{k}}_{-}\}|n^{\prime },m^{\prime }\rangle =\frac{\mu_{n,m}^{2}}{a^{2}}\delta_{n^{\prime },n}\delta_{m^{\prime },m}.$$

Taking into account the loss of translational invariance along the *z* direction by replacing the wave vector-component *k*_*z*_ by the operator -*i*∂_*z*_ it is therefore convenient to write the resulting expression for the element matrix in a symmetrized form

$$\frac{1}{2}\left(\langle j|{\hat{k}}_{z}|j^{\prime }\rangle +\langle j^{\prime }|{\hat{k}}_{z}|j\rangle \right)$$

where index *j* stands for the piecewise wavefunctions (17). The resulting integrals in *z*-direction are solved numerically.
